# The chinchilla as a novel animal model of pregnancy

**DOI:** 10.1098/rsos.161098

**Published:** 2017-04-26

**Authors:** Emmeli Mikkelsen, Henrik Lauridsen, Per Mose Nielsen, Haiyun Qi, Thomas Nørlinger, Maria Dahl Andersen, Niels Uldbjerg, Christoffer Laustsen, Puk Sandager, Michael Pedersen

**Affiliations:** 1 Department of Obstetrics and Gynaecology, Aarhus University Hospital, Palle Juul-Jensens Boulevard 99, 8200 Aarhus N, Denmark; 2 Comparative Medicine Lab, Department of Clinical Medicine, Aarhus University, Palle Juul-Jensens Boulevard 99, 8200 Aarhus N, Denmark; 3 MR Research Centre, Department of Clinical Medicine, Aarhus University, Palle Juul-Jensens Boulevard 99, 8200 Aarhus N, Denmark

**Keywords:** pregnant animal models, chinchilla, placenta, imaging, comparative biology

## Abstract

Several parameters are important when choosing the most appropriate animal to model human obstetrics, including gestation period, number of fetuses per gestation and placental structure. The domesticated long-tailed chinchilla (*Chinchilla lanigera*) is a well-suited and appropriate animal model of pregnancy that often will carry only one offspring and has a long gestation period of 105–115 days. Furthermore, the chinchilla placenta is of the haemomonochorial labyrinthine type and is therefore comparable to the human villous haemomonochorial placenta. This proof-of-concept study demonstrated the feasibility in laboratory settings, and demonstrated the potential of the pregnant chinchilla as an animal model for obstetric research and its potential usefulness for non-invasive measurements in the placenta. We demonstrate measurements of the placental and fetal metabolism (demonstrated *in vivo* by hyperpolarized MRI and *in vitro* by qPCR analyses), placental vessels (demonstrated *ex vivo* by contrast-enhanced CT angiography) and overall anatomy (demonstrated *in vivo* by whole-body CT).

## 1. Introduction

While the choice of animal model in biomedical studies should generally reflect the appropriateness of the animal to study the particular mechanism of interest, often the choice also depends on the resources available, such as cost, housing, animal welfare, regulatory requirements, ethical considerations and the presence of experts working with the model, as well as traditionalism [1,2]. All this is reflected in the August Krogh principle [3] where emphasis should be put on convenience of the selected animal model. There may be several species in a group to choose from but the most reasonable model has to be selected based on the general considerations listed above. However, in obstetric research, it also depends on parameters such as gestation period, number of fetuses per gestation and placental structure. For clinical translation, the human gestation period is about 280 days, carrying only one fetus in 97–98% of all pregnancies and the placenta is described as haemomonochorial with a villous pattern in the placental exchange area [4].

In this proof-of-concept study, we introduce the pregnant chinchilla (*Chinchilla lanigera*) as an animal model for obstetric research. We show that introduction of this model may respond to our needs for increased knowledge about the placental and fetal metabolism (in this study demonstrated in four chinchillas *in vivo* by measurements of the pyruvate metabolism using hyperpolarized MRI and *in vitro* by qPCR analyses), placental vessels (demonstrated in one chinchilla *ex vivo* by contrast-enhanced CT angiography) and overall anatomy (demonstrated in one chinchilla *in vivo* by whole-body CT). Thus, the feasibility of the pregnant chinchilla for non-invasive modalities was proven, allowing *in vivo* characterization of the chinchilla anatomy, placental structure, haemodynamic measures and metabolism.

### 1.1. Pregnant animal models for biomedical research

Data on extant mammalian adult body mass, neonatal body mass, gestation length and litter size were acquired from the PanTHERIA database [5]. Only species for which all four parameters were available were included, resulting in a final dataset of 909 species from 26 orders. For in-depth studies of scaling in reproductive physiology in animals, we refer to publications by Martin *et al.* [6,7].

**Figure 1.**
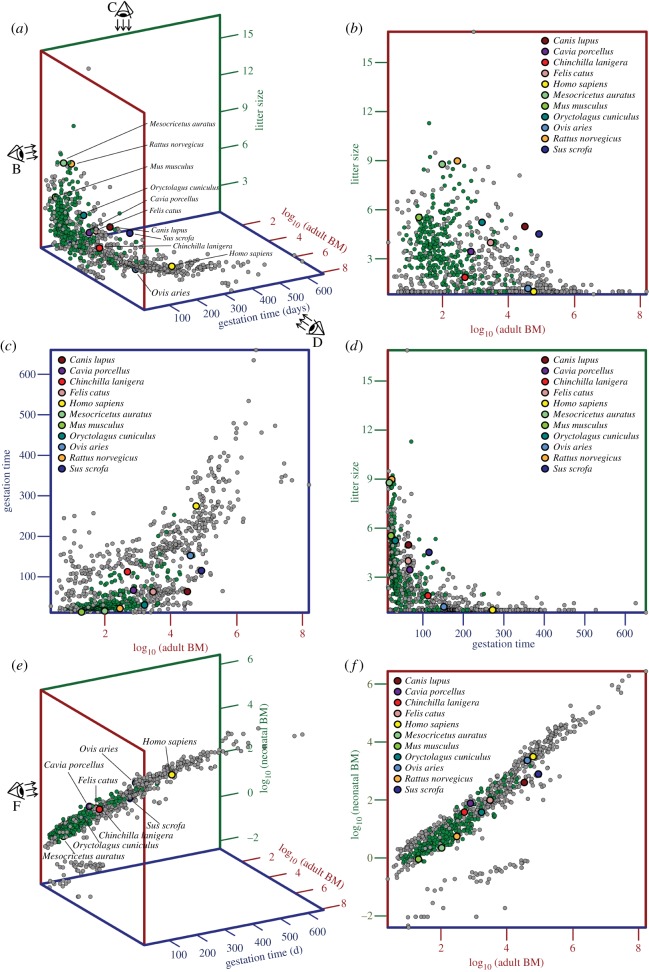
Reproductive physiology makes the chinchilla an appropriate model to study placental metabolism and function. (*a*) Three-dimensional scatterplot of log_10_ transformed adult body mass (BM), gestation time and litter size of 909 extant mammals. Rodents are plotted in green. Well-established research animals are highlighted. (*b*–*d*) Litter size plotted against log_10_(adult BM) (*b*), gestation time plotted against log_10_(adult BM) (*c*) and litter size plotted against gestation time (*d*). The chinchilla (red) has a small litter size and a long gestation time for its BM compared with established animal models. (*e*) Three-dimensional scatterplot of log_10_ transformed adult BM, gestation time and log_10_ transformed neonatal BM of 909 extant mammals. Rodents are plotted in green. Well-established research animals are highlighted. (*f*) log_10_(neonatal BM) plotted against log_10_(adult BM). The chinchilla (and the guinea pig) give birth to precocious offspring relative to their body size compared with all other mammals and especially other rodents.

The optimal animal model to study placental metabolism and function would combine the low maintenance cost of a rodent model with a small litter size that allows for individual fetus tracking over time and a long gestation period to increase temporal resolution in longitudinal studies. The reproductive physiology of the hystricomorph chinchilla is somewhat extraordinary compared with that of other non-hystricomorph rodent models typically applied in biomedical research, and these characteristics make the chinchilla very suited for placental studies. The litter size of 1–6 cubs is small for a rodent [8] (figure 1*a*,*b*,*d*) and very often chinchillas will carry only one offspring. Furthermore, the gestation period of 105–115 days [8] is long compared with other established rodent models, such as the guinea pig (59–72 days [9]), rat (21–23 days [10]), mouse (19–20 days [11]), hamster (15.5 days [12]) and even the approximately threefold larger lagomorph rabbit (29–35 days [13]; figure 1*a*,*c*,*d*). The relatively long gestation period allows higher temporal investigations of the placenta and the fetus, and the small litter size circumvents the challenge of pairing individual measurements on the placenta and fetus over time in longitudinal experiments. In addition, the body mass (i.e. size) of neonatal chinchillas is relatively large compared with the body mass of adults, compared with other rodent models, with the exception of the hystricomorph guinea pig, which also gives birth to precocious offspring (figure 1*e*,*f*). These proportions are beneficial in imaging studies, such as MRI and CT, in terms of spatial resolution and sensitivity. Besides, the long-tailed chinchilla may also have a clear advantage over the virtually tailless guinea pig regarding intravenous access.

### 1.2. Animal placentae: comparative characteristics

The main purpose of the placenta is to facilitate the exchange of oxygen, nutrients and waste products. Glucose is the main fetal energy source. Despite the large fetal demand for glucose, the placenta itself consumes about half of the supply of glucose, metabolizing and converting a great amount to lactate [14]. Lactate is delivered to the fetus, where it is used as an important substrate for fetal growth and metabolism [15]. Placental glucose transfer and metabolism have been studied for several years both *in vitro* and *in vivo* [16,17]. The placental uptake and metabolism of glucose depend on many different factors and determinants such as maternal supply, fetal demands, hormones, growth factors, cytokines, placental blood flow and placental size. An altered placental glucose metabolism can influence fetal growth and is found in pregnancies with, for example, intrauterine growth restriction (IUGR) [18–20], pre-eclampsia [21] and diabetes [22]. However, there is today insufficient knowledge about the pathological mechanisms in the placenta connected to these conditions, and an appropriate animal model for this purpose is warranted.

**Figure 2.**
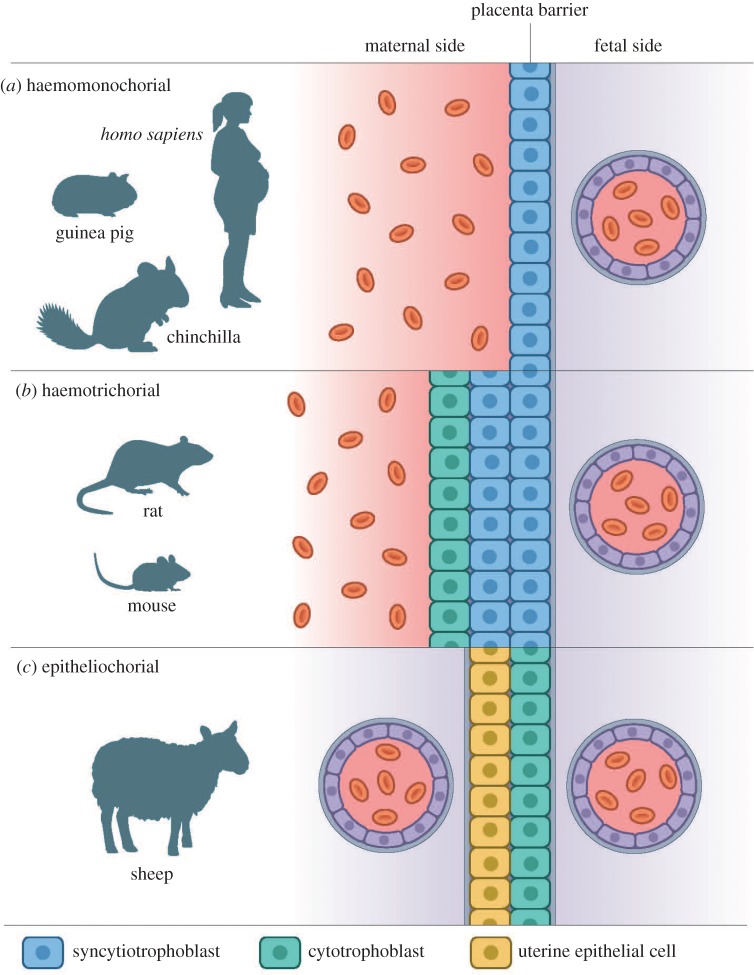
Schematic of different placenta barriers as seen in a microscope. (*a*) Haemomonochorial placenta barrier as seen in, for example, human, guinea pigs and chinchillas. Only one layer of syncytiotrophoblasts separates the maternal blood space from the fetal capillaries. (*b*) Haemotrichorial placenta barrier as seen in, for example, mice and rats. Three layers of trophoblast cells separate the maternal blood space from the fetal capillaries. (*c*) Epitheliochorial placenta barrier as seen in, for example, sheep. One layer of uterine epithelium cells and one layer of trophoblast cells separate maternal and fetal capillaries. Furthermore, in all three cases, maternal and fetal blood is separated by connective tissue and basal laminae.

On the histological level, the chinchilla placental structure is haemomonochorial of the labyrinthine type [23,24]. Haemomonochorial refers to the placenta barrier that consists of only a single layer of syncytiotrophoblasts dividing the maternal blood space from the blood in the fetal capillaries (figure 2*a*). The placentae in most rodents (rats and mice) are also haemochorial, but in contrast to the previously mentioned single-layered syncytiotrophoblast, they are haemotrichorial with two layers of syncytiotrophoblasts and one layer of cytotrophoblasts (figure 2*b*) [25,26]. However, the guinea pig placenta is also haemomonochorial of the labyrinthine type [4], and therefore, the pregnant guinea pig is today the most important animal model for placental studies in obstetric research [27].

Considering animal models larger than rodents, the sheep has a cotyledonary placenta, a long gestation period of about five months and gives birth to a single mature lamb with a weight comparable to the human newborn [1]. The pregnant sheep is therefore a good model for investigating fetal physiology, but it has shown limited value for translational placental research as the placenta has a diffuse gross morphology and is epitheliochorial without any trophoblast invasion in the uterine vessels (figure 2*c*) [28].

### 1.3. The chinchilla pregnant animal model: comparative characteristics

The chinchilla is a well-established animal model in biomedical research, mainly applied in the study of development, function and disease of the auditory system [29]. Owing to distinct similarities with humans in the anatomy and physiology of the middle and inner ear as well as the Eustachian tube, the chinchilla is considered an ideal model for a range of auditory diseases, in particular otitis media [30–32]. As such, the requirements for laboratory husbandry of chinchillas are well established [8] along with standard operating procedures for basic experimental methods such as appropriate animal handling, body fluid sampling and compound administration [33], paving the way for the use of the chinchilla in other fields in biomedical research. There are no inbred strains or breeds of domestic chinchillas; however, three different varieties are recognized: *la plata*, *costina* and *raton* with small differences in musculature and limb length in addition to different colour variants such as grey, white, black and brown [34]. An important contribution to the chinchilla as a research model was made in 2012, when whole-genome shotgun sequencing of the chinchilla genome was completed, and the following year, when the sequencing of the mitochondrial genome was completed [35]. Recently, the Chinchilla Research Resource Database (http://crrd.mcw.edu) has compiled this genomic information with available RNA sequencing data and established a user-friendly interface to retrieve and analyse genomic and transcriptomic chinchilla data and compare this with the human counterpart [36].

## 2. Material and methods

In order to demonstrate the usefulness of the pregnant chinchilla for placental and fetal research, we undertook *in vivo*, *in vitro* and *ex vivo* studies on the animal. A special advantage of *in vivo* measurements is the convenience that measurements can be performed many times during the gestation period. Furthermore, it is possible to measure the placental function while influenced by maternal and fetal factors, such as supply of hormones, metabolic substrates, growth factors, etc. [37]. In this study, *in vivo* imaging is demonstrated using hyperpolarized MRI; a non-harmful imaging modality using non-ionizing endogenous substrates for interrogating accumulation and metabolic pathways. Note, however, that hyperpolarized MRI is a relatively new technique, not yet introduced in clinical settings, but its clinical potential for non-invasive examination of the fetoplacental metabolism and nutrient transport without teratogen concerns is promising. In addition, imaging of the skeletal structure and placental vessels were visualized by standard CT procedures.

### 2.1. Chinchilla preparation

The pregnant chinchillas were purchased from approved dealers. In the last half of pregnancy, four animals were anaesthetized with 2% sevoflurane in 2 l atmospheric air as breathing gas. All animals were weighed and blood glucose levels measured from tail capillary blood with a Contour blood glucose meter. To prevent dehydration, the animal received a subcutaneous injection of 5 ml isotonic saline and 3 ml isotonic glucose after being anaesthetized. Tail vein catheterization was performed and an isotonic glucose infusion of 6 ml h^−1^ was administered for approximately 1 h to avoid a fasting state. The body temperature was measured by a rectal probe and maintained using a temperature-controlled surgical plate.

### 2.2. Hyperpolarized MRI

**Figure 3.**
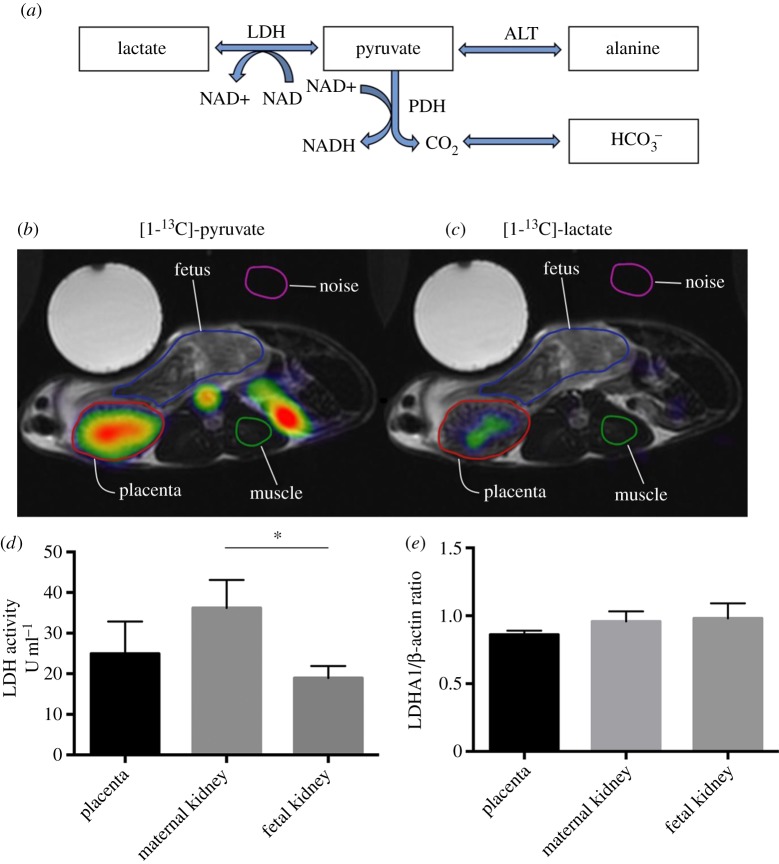
(*a*) The principle of hyperpolarized magnetic resonance imaging (MRI) using [1-^13^C]-pyruvate, which is a metabolite of glucose. [1-^13^C]-pyruvate is metabolized to [1-^13^C]-lactate, [1-^13^C]-alanine and ^13^CO_2_, which is in equilibrium with ^13^C-bicarbonate $\left({\text{H}}^{13}{\text{CO}}_{3}^{-}\right)$. These pathways are facilitated by the enzymes LDH, alanine transaminase and pyruvate dehydrogenase, respectively. (*b*,*c*) Representable ^1^H images with overlaid metabolic maps of [1-^13^C]-pyruvate, showing uptake primarily in placenta, while little signal is observed in the fetus (*b*), and metabolic conversion of [1-^13^C]-pyruvate to [1-^13^C]-lactate similarly localized to the placenta (*c*). Enclosed circles represent applied ROIs of the placenta, fetus, maternal skeletal muscle tissue and noise. SNRs for hyperpolarized [1-^13^C]-pyruvate in the placenta, fetus and maternal skeletal muscle tissue showed that hyperpolarized [1-^13^C]-pyruvate was significantly supplied and accumulated in the placenta compared with the fetus and maternal skeletal muscle tissue. (*d*) LDH activity in U ml^−1^, indicating a general high LDH activity in maternal renal tissue compared with fetal renal tissue (*n* = 4). (*e*) mRNA expression of lactate dehydrogenase (LDHA1), indicating no significant difference between placental tissue compared with maternal and fetal renal tissue (*n* = 4). Statistical comparisons among groups were performed with a one-way ANOVA using multiple comparisons with Fisher's test; *p < 0.05. Data are shown with mean + s.d.

In all animals, *in vivo* imaging of anatomy and placental pyruvate metabolism was performed with hyperpolarized MRI, as pyruvate, lactate, alanine and bicarbonate are fundamental intermediates in the metabolism of glucose (figure 3*a*) [38]. MRI was performed with a 3T system (GE Healthcare, Waukesha, WI, USA) equipped with a ^1^H 8-channel cardiac array coil for anatomical (T2-weighted) scans and a ^13^C Helmholtz loop coil (*ø* = 20 cm) for metabolic maps of endogenous substrates using hyperpolarized MRI. Hyperpolarized MRI is a method for assessing metabolic processes in tissues in real time. *In vivo* imaging of the chinchilla placenta was performed with standard ^1^H-MRI, allowing visualization of the fetus and placenta, and hyperpolarized ^13^C-MRI for assessment of pyruvate metabolism (figure 3*a*) [39]. Two millilitres of 90 mM [1-^13^C]-pyruvate were injected into the tail vein catheter over a period of 15 s. A slice-selective ^13^C IDEAL spiral MRI sequence was used, acquiring images every 5th second and initiated 30 s after the start of [1-^13^C]-pyruvate injection. The sequence had the following parameters: excitation flip angle = 15°, 11 IDEAL echoes and one initial spectrum per IDEAL encoding, TR/TE/ΔTE = 100 ms/0.9 ms/0.9 ms, FOV = 120 × 120 mm^2^, 5 × 5 mm^2^ real resolution and an axial slice thickness of 20 mm covering the placenta and a part of the fetus. The temperature, saturation and respiration were monitored during MRI, and the animal was heated with a temperature-controlled heater (SA Instruments, Stony Brook, NY, USA). After the last MRI scan, each chinchilla was euthanized using pentobarbituate, and it was secured that the fetus was dead. MRI analyses included regions-of-interests of placenta, fetus, maternal skeletal muscle tissue and noise that were manually drawn on the ^1^H anatomic images in OsiriX (The Osirix Foundation, Geneva, Switzerland, http://www.osirix-viewer.com) [40] and transferred to the ^13^C images (figure 3*b*,*c*). The ROI signal intensities were analysed according to signal-to-noise ratio, by dividing the sum of the pyruvate signals with the mean of the noise signal for each metabolite. The noise signal was calculated from a region outside the animal. Only one placenta and fetus was analysed in the chinchilla carrying two fetuses.

### 2.3. Chinchilla placental cellular activity

Following the last MRI scan, the placental metabolism was evaluated *in vitro* in each animal. Placental and renal tissues from the mother and the fetus were used for analysing the activity and mRNA expression of lactate dehydrogenase (LDH). For activity measurements, these tissues were instantly frozen in liquid nitrogen and stored at −80°C. LDH activity assay kits were used according to the manufacturer's instructions (Sigma-Aldrich, Copenhagen, Denmark). Briefly, tissue was homogenized and purified in LDH assay buffer. All analyses were performed on 96-well costar half area plates in a PHERAstar FS micro plate reader (BMG Labtech, Ortenberg, Germany). For qPCR measurements, RNA extraction was carried out using a NucleoSpin RNA II kit (Stratagene; AH Diagnostics, Aarhus, Denmark). From the isolated RNA, complementary DNA (cDNA) was synthesized using a RevertAid First strand cDNA synthesis kit (MBI Fermentas, Burlington, Canada). All procedures followed the manufacturer's instructions. One hundred nanograms of synthesized cDNA was used as a template for PCR amplification. qPCR was then performed using the SYBR Green qPCR Master Mix (Stratagene; AH Diagnostics) according to the manufacturer's instructions. Primer specificity of the products was verified by gel electrophoresis and melting curve analysis. The following primer sequences were used: LDHA1: sense 5′-GGT GGT TGA CAG TGC GTA TG-3′, antisense 5′-TCA CAA CAT CGG AGA TTC CA-3′. β-Actin: sense 5′-GAG ATG AAG CCC AGA GCA AG-3′, antisense 5′-CTG GGT CAT CTT CTC ACG GT-3′.

### 2.4. Computed tomography

Whole-body imaging and *ex vivo* placental contrast-based CT angiography were performed using a 64-slice Siemens Somatom Definition (Siemens Medical Solutions, Erlangen, Germany). One pregnant non-sedated chinchilla underwent a CT procedure with the following acquisition parameters: a slice collimation of 4 mm; a pitch of 2°; 32 rotations (resulting in a 25.6 cm scanning volume) and a matrix size of 512 × 512. Acquisition parameters using the Somatom Volume Zoom option included a slice collimation of 4 × 1 mm, a rotation time of 0.5 s, 5–8 mm table feed/rotation, a matrix size of 512 × 512 and scan duration of 25–30 s. Transverse images were reconstructed with a section thickness of 1.25 mm and were reconstructed at 0.6 mm intervals. One chinchilla was euthanized with pentobarbituate, and one placenta was harvested immediately after elective caesarean section and placed in a water bath at 37°C. The umbilical cord was cut off approximately 5 cm proximal to the insertion to the placenta. The amnion was cut off as well. The blood vessels of the umbilical cord were catheterized using three venous catheters. The venous catheters were fixed to the blood vessels using polyester sutures and the umbilical cord was clamped using a suture to prevent reflux of the injected solutions. The placenta was then perfused using saline with 5000 IU l^−1^ heparin [41]. The solution was heated to 37°C and injected into the two umbilical arteries with a pressure-controlled pump using the sphygmanometric principle, followed by adding gelatin and a CT contrast agent (Mixobar; Astra Tech, Mölndal, Sweden). The placenta was allowed to cool to room temperature and CT angiography was performed in a similar way as the whole-body CT protocol.

## 3. Results

Although the animals showed relatively unaffected behaviour following the interventions, we observed miscarriage in two pilot animals after the experimental procedure; a possible effect of the anaesthesia or other stress-related causes. In general, we found that the tail vein was hard to localize due to the dark skin of the chinchilla, and catheterization was challenging. However, heating the animal for 15 min before sedation facilitated an increased tail blood flow, expansion of the tail veins and allows an easier insertion of the catheter. Furthermore, we found that the chinchilla had to be given 2 l oxygen until full recovery during awakening to avoid coma and death.

### 3.1. Metabolism: hyperpolarized MRI and cellular activity

In total, we performed six MRI scans on four animals. Animal characteristics are presented in table 1, ensuring that all animals were stable during the experiments. Hyperpolarized MRI revealed a high signal of [1-^13^C]-pyruvate and its derivative [1-^13^C]-lactate in the placenta (figure 3*b*,*c*), but these signals were absent in the fetus. No signal was observed from the derivatives [1-^13^C]-alanine and ^13^C-bicarbonate in the placenta and the fetus. By comparing [1-^13^C]-pyruvate SNRs, hyperpolarized [1-^13^C]-pyruvate was significantly supplied to the placenta compared with the fetus and maternal skeletal muscle tissue (figure 3*b*,*c*). Furthermore, we observed a general high LDH activity in maternal renal tissue compared with fetal renal tissue, and no significant difference in mRNA expression of LDHA1 between placental tissue compared with maternal and fetal renal tissue (figure 3*d*,*e*).

**Table 1.** Animal characteristics for the days of MRI scan and tissue sampling. Gestational age (days), maternal weight (g), blood glucose levels right after induction of anaesthesia before the scan and right before awakening or euthanasia after the scan (mM), mean rectal temperature (°C), respiration frequency (breaths min^−1^), blood haemoglobin saturation (%O_2_), placental weight (g), fetal weight (g) and number of fetuses for all four animals.

	chinchilla 1	chinchilla 2	chinchilla 3	chinchilla 4
*first scan*				
gestational age	69	80	82	—
maternal weight	587.5	655.3	704.2	—
blood glucose before/after scan	9.9/9.2	11.6/5.1	4.8/9.6	—
temperature	33.6	36.1	34.1	—
respiration	44	40	49	—
saturation	94	100	99	—
*second scan and tissue sampling*				
gestational age	97	98	103	96
maternal weight	641.2	687.5	751.7	716.3
blood glucose before/after scan	6.4/10.6	11.7/3.1	—	6.3/16.1
temperature	35.4	34.6	—	35.7
respiration	47	46	—	52
saturation	98	100	—	96
placental weight	4.2	4.3	8.4	3.7
fetal weight	37.5	33.5	59.7	27.7
number of fetuses	1	1	1	2

### 3.2. Anatomy: CT

**Figure 4.**
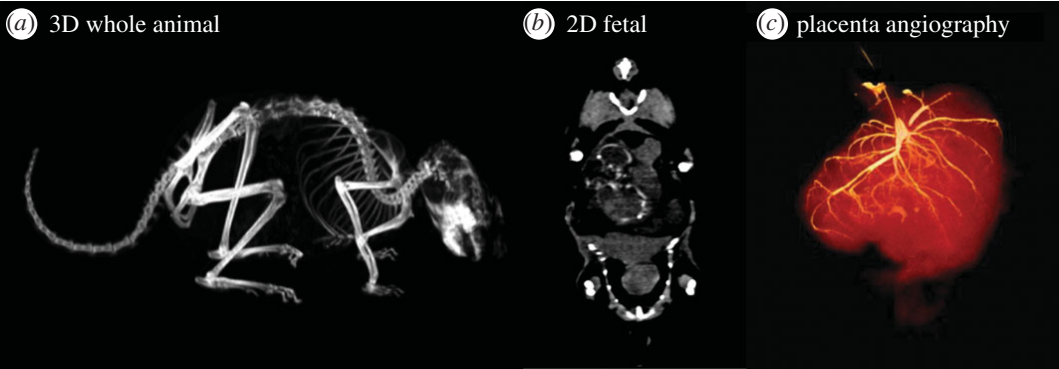
CT scan on whole chinchilla (*a*,*b*), demonstrating the low-radiopaque fetus, and CT-angiography of chinchilla placenta (*c*), showing a volume of the full placenta on 4000 mm^3^ and the fetal vasculature on 66 mm^3^ (more than 400 HU) and 193 mm^3^ (more than 250 HU).

Whole-body CT demonstrated the structural skeleton of the pregnant chinchilla (figure 4*a*). We observed that the fetus had a low radiopacity, resulting in low CT signal on the acquired three-dimensional CT images (figure 4*b*). The placenta was withdrawn for placental CT contrast-based angiography (figure 4*c*), demonstrating the characteristic higher-order branches of the fetal-placental vascular tree.

## 4. Discussion and conclusion

Although the closest models to humans are found among non-human primates, their endangered status and government sanctions limit the feasibility of these animals as models for research [27]. In obstetric research of non-primate models, three parameters are important when choosing the most appropriate animal model: the gestation period, number of fetuses per gestation and placental structure. In parallel, our understanding of the mechanisms involved in normal placental growth and function are limited, in part by the lack of imaging modalities that facilitate the study of both normal and abnormal pregnancy. Thus, this review and demonstration study revealed that the domesticated long-tailed chinchilla (*C. lanigera*) is a well-suited and appropriate model for these purposes. The pregnant chinchilla very often will carry only one offspring during pregnancy along with its relatively long gestation period of 105–115 days. Furthermore, the chinchilla placenta is of the haemomonochorial labyrinthine type and is therefore compatible to the human villous haemomonochorial placenta.

We demonstrated the potential of the chinchilla pregnancy model in obstetric research and its potential usefulness for MRI and CT measures in the placenta. Recently, a similar study by Friesen-Waldner *et al*. [42] tested the feasibility of hyperpolarized [1-^13^C]-pyruvate MRI for non-invasive examination of fetoplacental metabolism and nutrient transport in guinea pigs. In contrast to our proposed single fetal-placenta model, the Friesen-Waldner study used a multiple fetal-placenta model observing pyruvate and lactate signals in 30 placentae and fetal livers from seven guinea pigs. Our experiments corroborate the findings of significant amounts of [1-^13^C]-pyruvate and [1-^13^C]-lactate in the placenta; however, we observed no signal of these metabolites in the fetus during the 2 min of MRI acquisition (the time range in which the [1-^13^C]-pyruvate resides in a hyperpolarized state). Similar to our experiments, the Friesen-Waldner study did not observe any signal from [1-^13^C]-alanine or ^13^C-bicarbonate. Marković *et al*. [43] also used hyperpolarized [1-^13^C]-pyruvate to assess the placental metabolism in pregnant rats. They observed a slow build-up (starting at 16 s) and eventual decay (to 56 s) of the [1-^13^C]-pyruvate signal, and a weak ^13^C-alanine signal in the fetal livers. Fages *et al*. [44] used hyperpolarized ^13^C-urea and ^13^C-bicarbonate to monitor placenta-fetal perfusion and uptake on pregnant rats at late gestation stages (embryonic days 17–21). A generally slow perfusion/diffusion of ^13^C-urea and ^13^C-bicarbonate from the placenta to the fetus was observed. The lack of signal in our chinchilla fetuses, which is in contrast to these guinea pig and rat studies, may be the result of differences in the placental handling of these metabolites among species or difficulties in differentiating fetal tissue from surrounding tissue in multiple fetal-placenta models. Because the chinchilla only carries one to two cubs per pregnancy, we were able to acquire images with a higher resolution in order to distinguish the signals from the placenta and the fetus.

In this study, we demonstrated that the fetus and placenta of the pregnant chinchilla are hardly visible with conventional CT, but we found that the intraplacental vessel arrangement was revealed by CT angiography, allowing for quantitative evaluation of the fetal-placental vascular tree during pregnancy. In conclusion, this proof-of-concept study demonstrated the feasibility of the pregnant chinchilla as an alternative animal model compared with other rodent models typically employed in obstetric research, having reproductive characteristics that in part resemble those in the human. Thus, we believe that this model may in future contribute to characterization of the placental structure, function and metabolism in various pathological conditions during pregnancy, such as IUGR, pre-eclampsia and diabetes.

## Data accessibility

Dataset obtained for this study is available at the Dryad Digital Repository: (http://dx.doi.org/10.5061/dryad.9cp3j) [45].
